# Revision of the genus *Limobius*, with the description of a new species (Coleoptera, Curculionidae, Hyperini)

**DOI:** 10.3897/zookeys.709.14877

**Published:** 2017-10-18

**Authors:** Jiří Skuhrovec, Miguel A. Alonso-Zarazaga

**Affiliations:** 1 Group Function of Invertebrate and Plant Biodiversity in Agro-Ecosystems, Crop Research Institute, Drnovská 507, CZ – 161 06 Praha 6 – Ruzyně, Czech Republic; 2 Depto. de Biodiversidad y Biología Evolutiva, Museo Nacional de Ciencias Naturales (CSIC). José Gutiérrez Abascal, 2. E-28006, Madrid, Spain

**Keywords:** Check-list, Coleoptera, Curculionidae, description, distribution, Europe, Hyperini, key, *Limobius*, new species, Palaearctic Region, taxonomy

## Abstract

The new species, *Limobius winkelmanni* **sp. n.** is described, keyed, and illustrated. This enigmatic new species has seven desmomeres as other Hyperini-species, but according to shape of elytra and aedeagus, which are typical for representatives of *Limobius*, it is treated in this genus. The actualised key and check-list of *Limobius* is presented. The taxonomical position and status of the genus *Limobius* within the tribe Hyperini is also discussed here.

## Introduction

The phylogenetic and taxonomic position of hyperines is still unresolved. In the previous study, hyperines together with Bagoini and Gonipterini were regarded as unplaced tribes in Curculionidae (Oberprieler et al. 2014a). Before recent studies, the hyperines have traditionally been regarded as a subfamily of Curculionidae (e.g., Thompson 1992; Zimmerman 1992; Alonso-Zarazaga and Lyal 1999; Anderson 2002; Marvaldi et al. 2002; Marvaldi and Lanteri 2005; Bouchard et al. 2011); however, there are also some other opinions. Kuschel (1995) included them in his broad concept of Brachycerinae; Oberprieler et al. (2007) and McKenna et al. (2009) treated them as a tribe of Curculioninae; Legalov (2011a, 2011b) placed them as a tribe in Entiminae, although they do not share the autapomorphies of this subfamily (Oberprieler et al. 2014a); and finally, Hunt et al. (2007), Hundsdoerfer et al. (2009), McKenna et al. (2009), and Gunter et al. (2016) treated hyperines as at least belonging to a clade that included Entiminae, Cyclominae and Gonipterini.

Their only unique feature appears to be the specific meshed cocoon spun by the larvae from strands of protein secreted by the Malpighian tubules (Scherf 1964; Kenchington 1983), and ectophagous larvae (Oberprieler et al. 2014a; Skuhrovec and Bogusch 2016). Petri (1901) was the last to define this group using ten morphological characters. However, of these, only the features of the trochanters, claws, and pygidium are diagnostic, and none of these are unique to Hyperini (Oberprieler et al. 2014a). Petri (1901) divided the Hyperini into two subtribes, Hyperina and Cepurina, based on the shape of the mesepimera and the length of the metanepisterna and the relative width and angle of their junction with the mesepimera. Legalov (2007, 2010, 2011) divided this tribe into five subtribes: Cepurina, Hyperina, Coniatina, Macrotarrhusina and Phaeopholina, based on several morphological characters, but such a distinction requires a more comprehensive study of the whole tribe and is equally unlikely to yield meaningful synapomorphies to identify family group taxa within the group (Oberprieler et al. 2014a). Hyperini currently comprises approximately 44 genera and 500 described species (Oberprieler et al. 2014a). Skuhrovec (unpublished data, see Skuhrovec and Bogusch 2016) recently divided this tribe into three “operating” groups with different distributions: (1) the Palaearctic region (Hyperina) – the majority of the species (ca. 370) occur in the Palaearctic region, with far fewer species in the Nearctic (ca. 20); (2) the circumtropical region (Cepurina) – occurs in the Neotropical (ca. 40), Afrotropical (16) and only two in the Oriental region; and (3) the Australian/Pacific region (Australian Hyperini and *Phaeopholus* Roelofs, 1873) – occurs in the Australo-Pacific region with ca. 45 species (Oberprieler et al. 2014a).

Only the Palaearctic fauna of Hyperini have received recent taxonomic attention. Skuhrovec (2005a, 2005b, 2006a, 2006b, 2007) and Skuhrovec and Bogusch (2016) studied the larvae of *Donus* Jekel, 1865, *Hypera* Germar, 1817 and *Metadonus* Capiomont, 1868, clarified (2008) the complex nomenclature of the large and important genera *Brachypera* Capiomont, 1868, *Donus* and *Hypera*, and revised (2012) the genus *Metadonus*. Alonso-Zarazaga and Lyal (2002) transferred the monobasic genus *Herpes* Bedel, 1874, previously classified in Brachycerinae or Rhythirrinini but in Thecesternini by Alonso-Zarazaga and Lyal (1999), to Hyperini. Legalov (2011a) recently upgraded a number of subgenera of *Coniatus* Germar, 1817, *Hypera* and *Macrotarrhus* Bedel, 1906 to generic status, but these taxonomic acts were published without detailed justification, and not all of the subsequent new combinations were given (Skuhrovec 2013a). Hence, Skuhrovec (2013a) and Oberprieler et al. (2014a) did not accept these taxonomic changes. A detailed comparative study of all hyperine is necessary in context of taxa representing all genera / subgenera and also species groups because most characters of adults and also of immature stages are based only on the relatively well-studied genera (Oberprieler et al. 2014a; Skuhrovec and Bogusch 2016).

The genus *Limobius* is one of the smallest genera, currently including only three known species. All known *Limobius* species develop on Geraniaceae plants (Smreczyński 1968, Koch 1992). *Limobius borealis* (Paykull, 1792) develops in the unripe flower heads of *Geranium* species instead as typical ectophagous Hyperini larva developing on the leaves or flowerhead. *Limobius*-species have only six desmomeres, and that is the only main character for differential distinction from *Hypera*-species, which have always seven desmomeres. Some weevil specialists consider *Limobius*-species only as small *Hypera* and believe that it is only at most a subgenus of *Hypera* based on the variability of this character (the number of desmomeres) in some other weevil groups (e.g. *Tychius* (Caldara 1990), *Ceutorhynchus* (Colonnelli 2004), *Onychapion* (Alonso-Zarazaga 1990), *Corimalia* (Schön and Skuhrovec 2016)). Despite this enigmatic differential character, *Limobius* was always strictly presented as a separate genus.

In this paper, *Limobius winkelmanni* sp. n. is described and illustrated. The first author received these specimens from the second author as a probably new species of unknown affinities, and this new species presented a taxonomic mystery which took ten years to solve. This enigmatic new species has seven desmomeres as other Hyperini species, but according to the shape of the prominent humeri and of the aedeagus, which are typical for representatives of *Limobius*, it is treated here as a member of the *Limobius*. The taxonomic position and status of *Limobius* within the tribe Hyperini are also discussed here.

## Materials and methods

**Taxonomy and photographic documentation.** Body lengths of all specimens were measured in dorsal view from the anterior border of the eyes to the apex of the elytra, excluding the rostrum. All measurements were measured in dorsal view. Dissected male and female genitalia were studied in glycerine and thereafter mounted dry on the same card as the respective specimen. Photos of genitalia were made using an Olympus BX40 microscope and combined in Zerene Stacker and GIMP2 software. Photos of adults were made with a Camera Canon Powershot A640 and Canon EOS 550D with a macro-objective MP-E 65 mm and combined using CombineZM and GIMP2 software. The terminology of the rostrum and the genitalia follows Oberprieler et al. (2014b).

**Specimen depositories and citations.** Specimens are deposited in the following museums and private collections:

**HWIC** Herbert Winkelmann private collection, Berlin, Germany;

**JSKC** Jiří Skuhrovec private collection, Praha, Czech Republic;

**MNCN** Museo Nacional de Ciencias Naturales (CSIC) in Madrid;

**TGAC** Tomasz Gazurek private collection, Warszawa, Poland.

Label data are cited in the description, separate lines on labels are indicated by “/” and separate labels by “//”.

## Taxonomy

### Genus Limobius

Taxon classificationAnimaliaColeopteraCurculionidae

Schoenherr, 1843

Figs 1 , 2–5 , 6–10 , 11–12 , 13–14

Limobius Schoenherr, 1843: 460 (original description)
Limobius : Capiomont (1868): 244 (monography); Petri (1901): 192 (monography); Winkler (1932): 1582 (catalogue); Csiki (1934): 54 (catalogue); Hoffmann (1954): 616 (fauna); Smreczyński (1968): 92 (fauna); Angelov (1978): 203 (fauna); Kippenberg (1983): 153 (fauna); Alonso-Zarazaga and Lyal (1999): 188 (catalogue); Morris (2002): 63 (fauna); Skuhrovec (2009): 3 (key); Skuhrovec (2013b): 435 (catalogue); Oberprieler et al. (2014a): 464 (handbook/catalogue).

#### Type species.

*Curculio dissimilis* Herbst, 1795: 290 (= *Curculio borealis* Paykull, 1792: 57).

#### Diagnosis.

Body 2.5–4.6 mm; entire body densely covered with appressed scales of different shapes, from scales divided into two lobes to base up to entire scales. Eyes elliptical to oval. Rostrum long to very long, narrow; in dorsal view distinctly longer than its base width (ratio more than 3.00); enlarged anteriorly, tapered to basal third part and afterward almost parallel-sided; in side view slightly curved; as long as pronotum (ratio = 0.95–1.10). Antenna with 6 or 7 desmomeres. Pronotum distinctly wider than long, widest at middle. Elytra with very distinct prominent humeri. Apex of penis enlarged, sometimes partially to the tip, and always without projecting setae. Apodeme of sternite VIII in females relatively long, with distinct long lateral arms; plate wide, not very well sclerotized, upper part not connected and bearing apically many distinct setae.

#### Biology.

These weevils occur in warm and dry habitats (calcareous hillsides, vineland, steppe, sandy habitats, meadows, clearings), and in mesophilic or moderately damp habitats of floodplains and hillsides (natural meadows) (Skuhrovec 2009). *Limobius* species develop on plants of two genera: *Geranium* and *Erodium* (all Geraniaceae) (Koch 1992; Skuhrovec 2009). The larvae do not develop on leaves as it is typical for Hyperini Marseul, 1863, but in the inner parts of the floral stalk. The main reason in this different strategy of Hyperini larvae is probably the size of the larva and is probably shared by other small species of Hyperini as it is known for *Hypera nigrirostris* (Fabricius, 1775).

#### Distribution.

The genus *Limobius* is mostly distributed in the western part of Europe and North Africa. Two taxa are known only from southern France. The only widespread taxon is *L. borealis borealis*, distributed in the whole western Palaearctic region, from Portugal to North Africa and eastwards to Iran (Skuhrovec 2013b).

### Limobius winkelmanni sp. n.

Taxon classificationAnimaliaColeopteraCurculionidae

http://zoobank.org/5E069633-7DAA-48F8-A10D-C310FB8EA232

**Figure 1.**
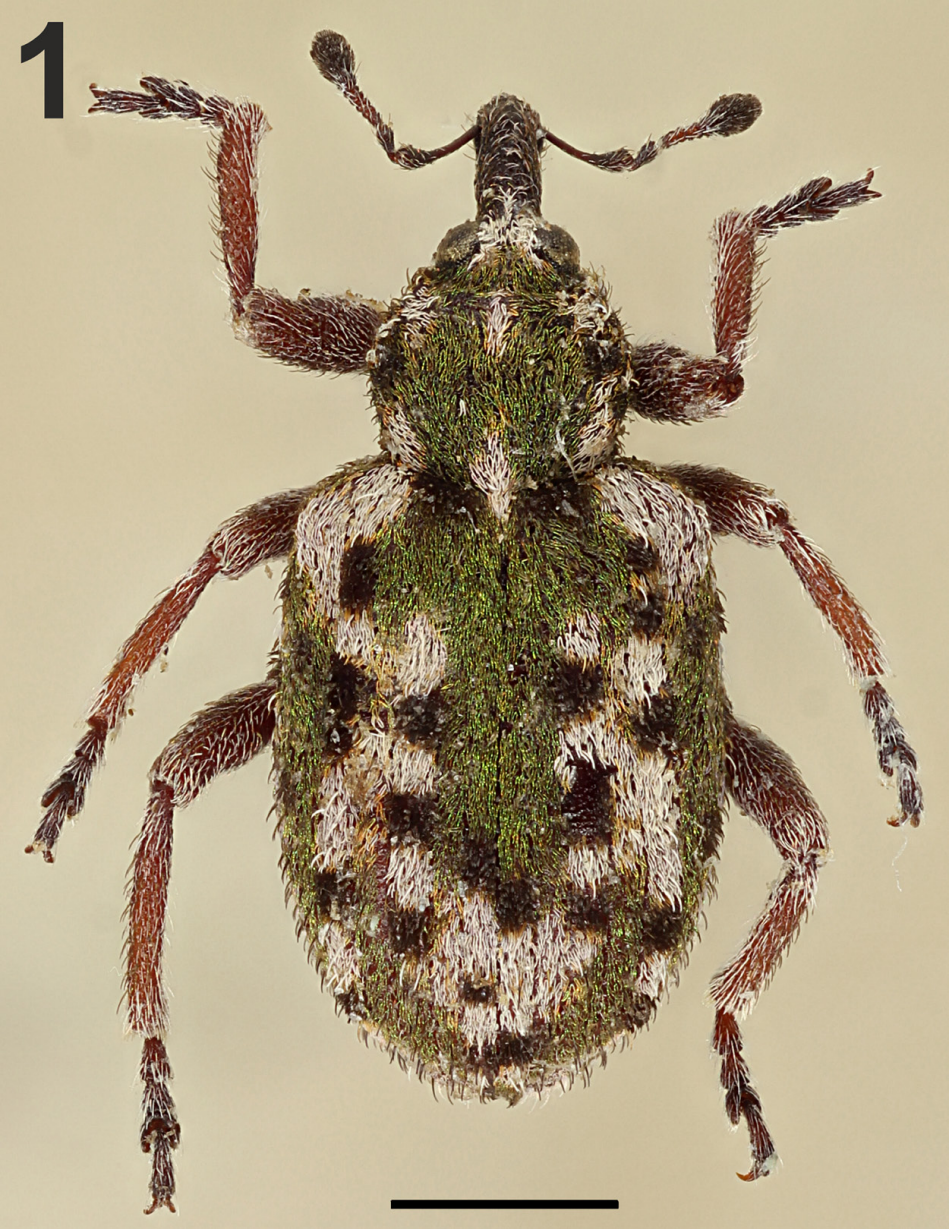
*Limobius winkelmanni* sp. n., habitus, male, Holotype, dorsal view. Scale bar 1 mm.

**Figures 2–5.**
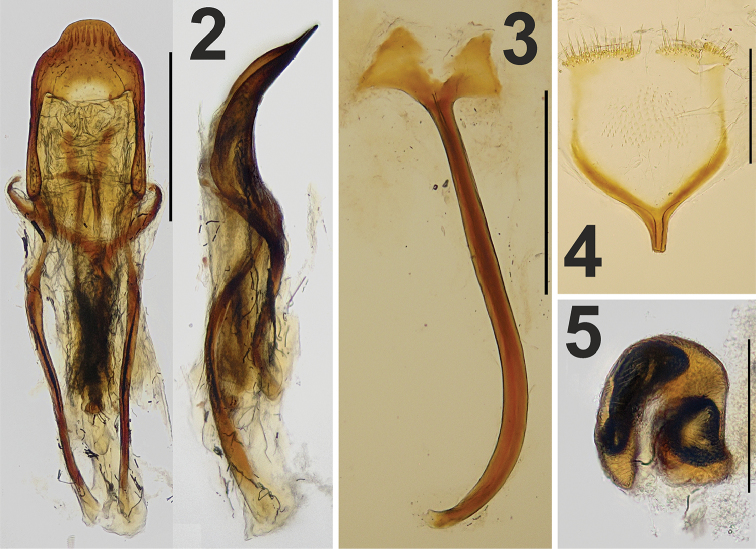
*Limobius winkelmanni* sp. n., male genitalia: **2** Aedeagus, dorsal, and lateral view **3** Spiculum gastrale; and female genitalia: **4** Spiculum ventrale **5** Spermatheca. Scale bars 0.5 mm (**2, 3**) and 0.2 mm (**4, 5**).

**Figures 6–10.**
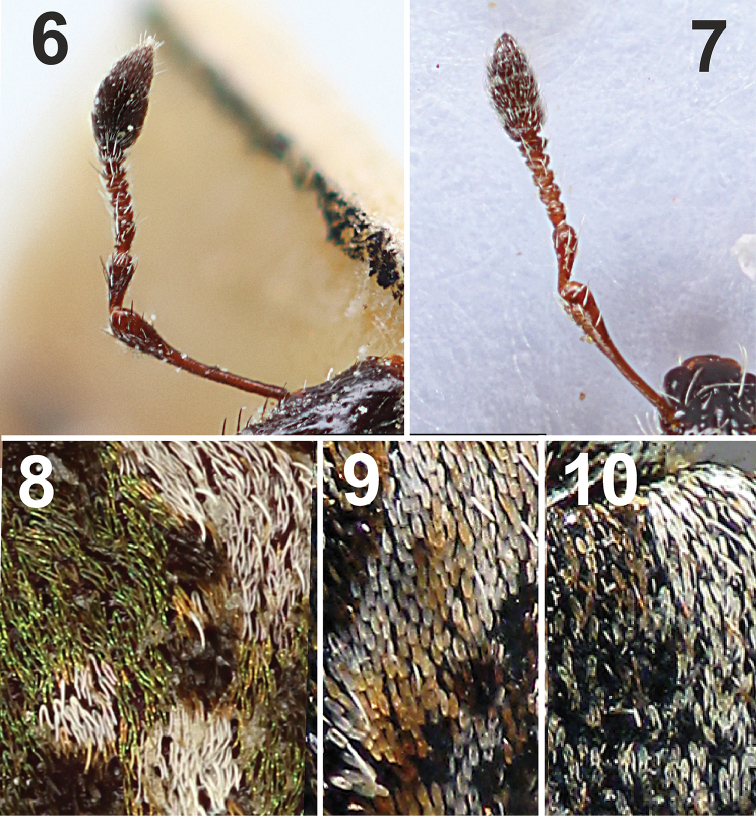
Morphological detail of *Limobius* species. **6** Antenna of *L. winkelmanni* sp. n. **7** Antenna of *L. borealis borealis* (Paykull, 1792) **8** Elytral scales of *L. winkelmanni* sp. n. **9** Elytral scales of *L. mixtus* **10** Elytral scales of *L. borealis borealis* (Paykull, 1792).

Figs 1 , 2–5 , 6 , 8

#### Type locality.

Altos de San Juan near El Escorial (Spain, limit between the provinces of Madrid and Ávila, 40°37'33.92"N 4°8'29.45"W).

#### Material examined.

Holotype ♂: ‘Escorial / Puerto [printed label] // Altos de / San Juan [handwritten label] // Altos de S. Juan / debajo de pequeñas / piedras con terreno / de esta composición [translation: Altos de S[an] Juan (S. = San), under small stones with a ground of this composition. i.e., a stony or gravelly ground] [handwritten label]’ (MNCN). Paratypes: 5 ♂♂, 12 ♀♀, ‘Escorial / Lauffer [printed label]’ (all MNCN; 1 ♂, 1 ♀ JSKC; 1 ♂, 1 ♀ HWIC); ‘ESPAÑA / P.M. de Moncayo / 02.04.2006 / leg. T. Gazurek [printed label]’ (1 ♂ TGAC). Specimens of the newly described species are provided with one red printed label: Holotype [or Paratype] / *Limobius* / *winkelmanni* sp. nov. / J. Skuhrovec & / M. Alonso-Zarazaga design. 2017.

#### Description

(Figs 1–6, 8). Colour of body integument vestiture reddish, light brown to brown, head, rostrum, all tarsomeres, distal parts of scape and desmomeres and the whole club black. Head, rostrum and antennae with sparse, very short, erect pale setae (distinctly shorter than claws). Frons covered with pale setae and elongated scales divided in two lobes apically. Vertex covered with green scales divided in two lobes to basal third of their length. Rostrum without distinct punctation, pale setae sparser than on frons. Pronotal vestiture dark reddish to brown, covered with pale setae and pale, green, reddish and light brown scales, all scales divided into two lobes to basal third of their length, and forming following colour pattern (Fig. 1): pale setae and scales in lateral lines and also two pale spots on disc of pronotum, first at apical margin and second at basal part; apical pronotal margin between pale lines and pale spot reddish to brown; green scales forming an H in middle part; two spots of black scales on the pale lines in the apical pronotal margin. Elytral vestiture reddish to brown, covered with scales divided in two lobes reaching their base, elytral intervals with pairs of pale and black; long, erect setae; scale colour pattern (Fig. 1): majority of dorsum with white setae and scales; black scales forming dark spots on basal part and afterwards white scales form white spots; green scales in the middle part in forming a T-shaped spot, lateral lines and a U-shaped spot in apical part (for detail see Fig. 1). Scutellum covered with white scales. Femora light brown to brown with pale and reddish to black long setae. Tibiae light brown to brown, bearing stout pale bristles apically. Tarsi dark reddish to black, with pale long setae, dark reddish parts with black spot in the middle, underside of first three segments on all tarsi with sparse small projecting scales (“soles”). Claws reddish to dark brown. Abdomen reddish to brown with long pale setae and a few scales divided in two lobes reaching their base.

*Head* (Fig. 1). Eyes elliptical to oval; upper margin higher than base of rostrum in lateral view, distinctly convex and bulging, distinctly wider than base of rostrum, ventral apex narrower than dorsal. Narrowest forehead distance slightly narrower as width of rostrum base. Head (occiput, vertex and forehead) without distinct punctation. Rostrum long, narrow; distinctly longer than its base width (ratio = 4.00); enlarged anteriorly to basal third part and afterward almost parallel-sided; in side view slightly curved; as long as pronotum (ratio = 0.95–1.10); scrobe distinct and deep; in dorsal view poorly visible, and only at antennal insertion; in lateral view distinct, slightly enlarged towards eyes, directed towards lower part of eye and not reaching them, near base of rostrum hardly noticeable; in front of antennal insertion broad, short and well visible. Occiput distinct.

*Antennae* (Figs 1, 6) connected to rostrum in apical quarter, long, slender. Scape narrow and elongate, slightly shorter than funicle (ratio to funicle = 0.8; ratio to funicle and club together = 1.2), almost reaching margin of eyes, slightly sinuous and abruptly widened apically. Funicle 7-segmented; desmomere 1 triangular, almost twice as long as 2; desmomere 2 also triangular; desmomeres 3–6 oval, slender, slightly widened at the apex; desmomere 2 twices longer than funicle segments 3–6, only slightly longer than 5 to 7 together, desmomeres 3 to 7 distinctly wider than long. Club elongate, 3-segmented, basal segment triangular, central segment rectangular, and apical segment also triangular, slightly longer than wide.

*Pronotum* (Fig. 1) distinctly wider than long (ratio = 1.20–1.35), widest at middle; anterior margin nearly straight in dorsal view; sides distinctly rounded; posterior margin 1.4 times longer than anterior margin; basal constriction noticeable, lacking distinct protuberances, slightly curved, distinctly visible from side and also dorsal view; without punctures.

*Elytra* (Fig. 1) almost rectangular, distinctly longer than wide (ratio = 1.35–1.47, see *Sexual dimorphism*), with base distinctly wider than the widest part of pronotum, with distinct and prominent humeral angles; basal margin distinctly curved; sides slightly convex, apically rounded. Elytral striae form 10 distinct rows, not visible because whole elytral surface covered by scales. Elytral intervals slightly prominent, and distinctly wider than striae.

*Mesoventer.* Mesoventral process narrow, not visible in lateral view.

*Legs.* Femora slightly inflated at middle; profemora almost as wide as rostrum; mesofemora and metafemora slightly slenderer. Tibiae apically widened. Meso- and metatibia straight, protibia slightly curved outwards. All tarsi similar; tarsomere 1 elongated, almost two times longer than tarsomere 2; tarsomere 2 almost squared, slightly widened at apex; tarsomere 3 triangular, distinctly bilobed almost to base; tarsomere 5 distinctly longer than tarsomere 1, slightly widened in apex. Claws free (not connate at base).

*Abdomen.* Abdominal ventrites 1–2 approximately of the same length, but twice the length of each abdominal ventrite 3 or 4; abdominal ventrite 5 almost of the same length as abdominal ventrites 1–2. Suture between abdominal ventrites 1 and 2 distinctly sinuous medially and shallow; other sutures straight, wide and deep.

*Sexual dimorphism.* Females slightly larger with more rectangular elytra (ratio length to wide of elytra = 1.4) than males (ratio = 1.35). Protibiae incurved in males and nearly straight in females. Abdominal ventrite 1 with a distinct depression in males but not in females. Apical abdominal ventrite with shallow medial impression in males. No differences in ratios of rostral length, pronotal length and width.

*Male genitalia.* Penis (Fig. 2) with tube in dorsal view sharply narrowed from base to basal 1/5, basal 2/5 tapered to previous width, then 1/5 almost parallel-sided; last 1/5 distinctly and triangularly narrowed towards rounded apex, in lateral view strongly curved in basal third, then parallel-sided and in apical third again strongly curved (Fig. 2). Temones more than one and a half as long as tube of penis. Spiculum gastrale (Fig. 3) stick-shaped, distinctly curved and subequal in length to half-length of penis; basal plate divided and triangular.

*Female genitalia.* Apodeme of sternite VIII relatively long, with distinct long lateral arms; plate starting near apical fifth of apodeme, at apex Y-shaped (Fig. 4); plate wide, not very well sclerotized, apical part not connected and bearing many distinct setae. Spermatheca (Fig. 5) C-shaped, with relatively elongated and curved cornu; ramus and nodulus short and strong.

#### Variation.

*Limobius winkelmanni* sp. n. is variable in body length: 4.2–4.6 mm (length of the holotype 4.4 mm). Colouration of pronotal and elytral vestiture may vary partially (see Description). No genitalic variations were observed.

#### Differential diagnosis.

This species is absolute unique not only in this genus, but also in whole tribe Hyperini. The species is characterized by the antenna with seven desmomeres (Fig. 6), a specific unusual colouration of vestiture (Fig. 1), the elytral scales divided in two lobes reaching their base (Fig. 8), and the body size more than 4 mm.

#### Etymology.

The new species is named after a close friend of the authors, Herbert Winkelmann (Berlin, Germany), who mentored the first author in Hyperini taxonomy and biology.

#### Bionomics.

Unfortunately, the host plant or any other biological data is not known. Weevils were collected probably at the beginning of the 20th century. We know only an exact locality of this weevil: Alto San Juan near Escorial – ca 35 km NW from Madrid, which is located in the mountains, 1734 m a.s.l. All known *Limobius* species develop on plants of the family Geraniaceae, but we cannot be sure if this will also be true with this new species. Additionally, its larval strategy could be different (see Introduction). *Limobius borealis* develops in the unripe flower heads of *Geranium* species instead as typical ectophagous Hyperini larva on the leaves or flowerhead. The main reason in this different strategy of Hyperini larvae is the size of larva, and *Limobius winkelmanni* sp. n. is distinctly larger than all *Limobius* species. However, its body length is still similar in size to some small *Hypera* species (e.g., *H. nigrirostris*), whose development is also in the unripe flower heads.

#### Distribution.

Central Spain (provinces Madrid and Zaragoza).

### Key to the species of the genus *Limobius*

**Table:** 

1	Desmomeres 7 (Fig. 6). Elytra mainly with green coloration (Fig. 1). Elytral scales divided in two lobes reaching their base (Fig. 8). Size 4.2–4.6 mm	***L. winkelmanni* sp. n.**
–	Desmomeres 6 (Fig. 7). Elytra mainly with brown coloration (Figs 11–14). Elytral scales not divided in two lobes reaching their base (Figs 9, 10). Size 2.5–3.5 mm	**2**
2	Elytral scales entire, not divided in two lobes (Fig. 9). Elytra with a transverse black stripe medially at midlength, posterior to this stripe with large whitish area lacking dark spots (Fig. 13)	***L. mixtus***
–	Elytral scales divided in two lobes apically, the emargination reaching at least midlength of each scale (Fig. 10). Elytra without a transverse black stripe medially at midlength, and also without large whitish area lacking dark spots (Figs 11, 12, 14)	**3**
3	Elytra only with a few projecting setae. (Fig. 12)	***L. borealis arvernus***
–	Elytra with numerous projecting setae (Figs 11, 14)	
4	Pronotum widest behind midlength, near to base; lateral stripe of scales on each margin yellow (Fig. 14). Apex of elytra U-shaped, distinctly rounded. Size: 3.5 mm (Type) (Fig. 14)	***L. dureti***
–	Pronotum widest at midlength, lateral stripe of scales on each margin white (Fig. 13). Apex of elytra V-shaped, gradually narrowing. Size: 2.5–3 mm (Fig. 13)	***L. borealis borealis***


**Figures 11–12.**
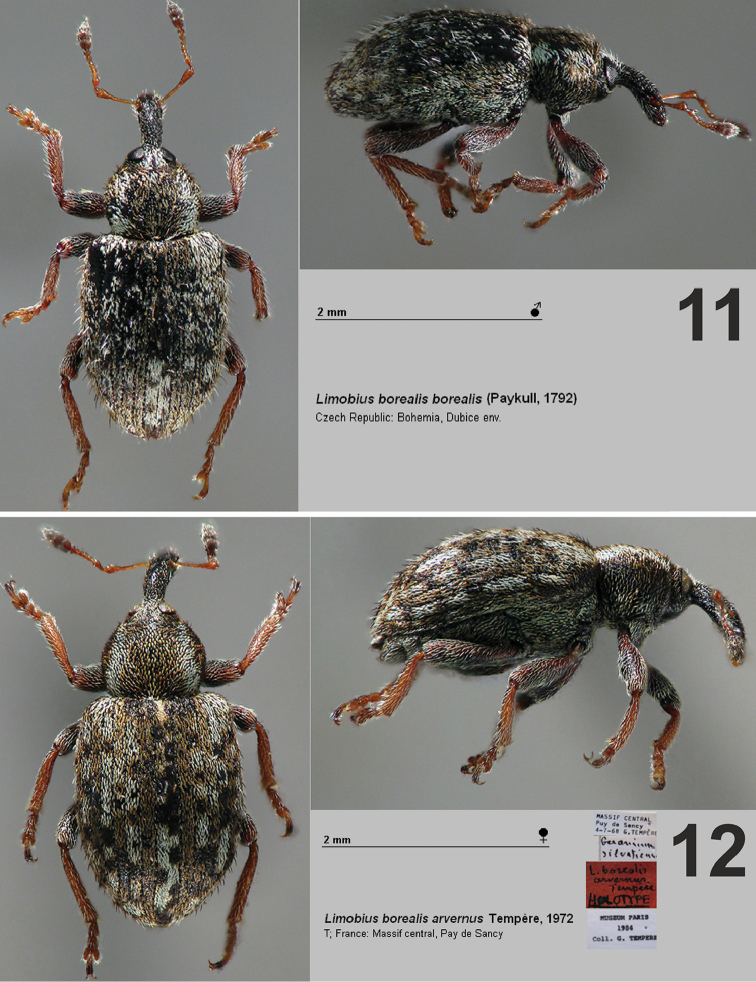
Dorsal and lateral view of *Limobius* species. **11** *L. borealis borealis* (Paykull, 1792) **12** *L. borealis arvernus* Tempère, 1972.

**Figures 13–14.**
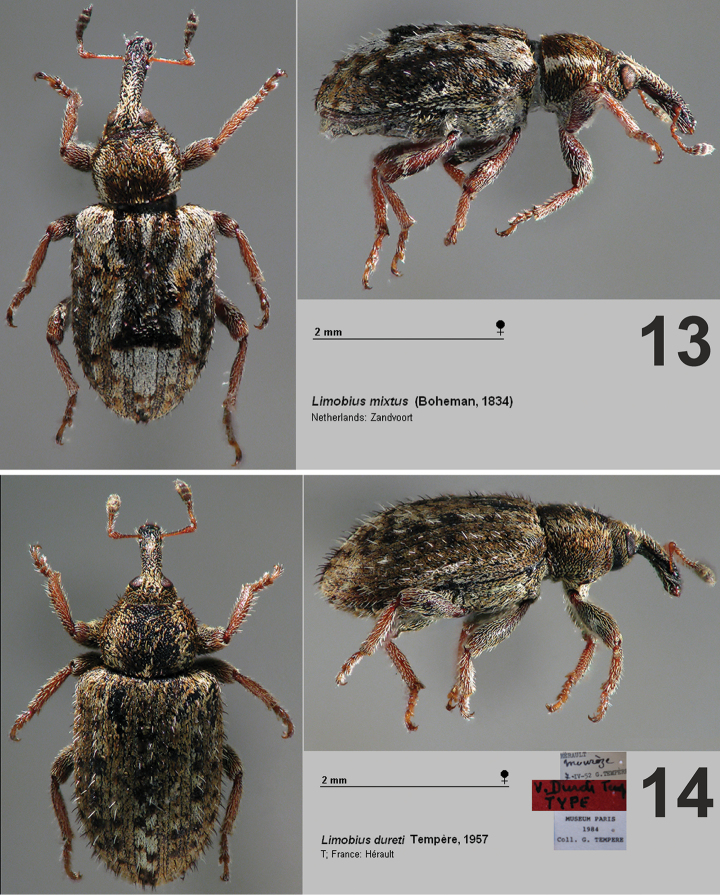
Dorsal and lateral view of *Limobius* species. **13** *L. mixtus* (Boheman, 1834) **14** *L. dureti* Tempère, 1957.

### Check-list of *Limobius* species

*L. borealis borealis* (Paykull, 1792) western Palaearctic region

*L. borealis arvernus* Tempère, 1972 southern France

*L. dureti* Tempère, 1957 southern France

*L. mixtus* (Boheman, 1834) western Europe, Malta; Africa: Morocco, Libya

*L. winkelmanni* sp. n. central Spain

### Taxonomic assignment and differential diagnosis of the genus

Whereas identification of the species is, in contrast to the majority of other Hyperini genera, quite easy, recognition of the genus *Limobius* within the tribe Hyperini has recently become a rather difficult matter. Hitherto, the number of desmomeres has been the only one mentioned differential character between genera *Hypera* and *Limobius*. This enigmatic new species *L. winkelmanni* sp. n. has seven desmomeres as other Hyperini-species, but the prominent humeral angles and the shape of the apical part of penis, which are typical of representatives of *Limobius*, compels us to place it in the genus *Limobius*. Consequently, the taxonomical position and status of *Limobius* within the tribe Hyperini has also to be discussed here.

The character of the number of desmomeres has high variability also within different genera in many weevil groups (e.g. *Tychius* (Caldara 1990), *Ceutorhynchus* (Colonnelli 2004), *Onychapion* (Alonso-Zarazaga 1990), *Corimalia* (Schön and Skuhrovec 2016)), and the majority of weevil specialists (included the first author) suspect that the genus Limobius should be a subgenus of Hypera. The discovery of *L. winkelmanni* sp. n., supports this opinion. However, the preliminary molecular studies in two independent data sets (Skuhrovec and Alonso-Zarazaga, unpublished data) have produced variable results. In both studies, *Limobius* species are sister to the Hyperini branch and suggests that *Limobius* should be considered a lineage separate from *Hypera*. Given the preliminary results, it is premature to state that *Limobius* is a primitive, relict group. The development of *Limobius* immature stages also partly support this hypothesis due to larval development inside the unripe flower heads as it is more typical for weevils than pure ectophagy. On the other hand, some small *Hypera* species (e.g., *H. nigrirostris*) have an identical developmental niche as *Limobius* species (in the unripe flower heads), which could be the evolutionary origin of the ectophagy present in the other groups of Hyperini.

Taxonomic positions and relatives of genera, subgenera and species-groups within the tribe Hyperini (including presently three apparently monophyletic groups, the Holarctic Hyperina with ca. 400 species, the circumtropical Cepurina with ca. 50, and the Australian/Pacific unnamed group with ca. 45 species) are completely unknown, and only a detailed morphological revision of the whole group and a molecular phylogeny may resolve these problems. The status of several genera and subgenera is very weak and the complete revision of this still unapproachable tribe, as well as a new evaluation of the characters used for the genus *Limobius* by Petri (1901), is necessary.

## Supplementary Material

XML Treatment for Limobius

XML Treatment for Limobius winkelmanni
